# The combination of electroporation and electrolysis (E2) employing different electrode arrays for ablation of large tissue volumes

**DOI:** 10.1371/journal.pone.0221393

**Published:** 2019-08-22

**Authors:** Nina Klein, Enric Guenther, Florin Botea, Mihail Pautov, Simona Dima, Dana Tomescu, Mihai Popescu, Antoni Ivorra, Michael Stehling, Irinel Popescu

**Affiliations:** 1 Inter Science GmbH, Lucerne, Switzerland; 2 Institut fur Bildgebende Diagnostik, Offenbach, Germany; 3 Department of Information and Communication Technologies, Universitat Pompeu Fabra, Barcelona, Spain; 4 Center of General Surgery and Liver Transplantation–Fundeni Clinical Institute, Bucharest, Romania; 5 Center of Translational Medicine–Fundeni Clinical Institute, Bucharest, Romania; 6 Department of Anesthesiology and Intensive Care 3, Fundeni Clinical Institute, Bucharest, Romania; Consiglio Nazionale delle Ricerche, ITALY

## Abstract

**Background:**

The combination of electroporation with electrolysis (E2) has previously been introduced as a novel tissue ablation technique. E2 allows the utilization of a wide parameter range and may therefore be a suitable technology for development of tissue-specific application protocols. Previous studies have implied that it is possible to achieve big lesions in liver in a very short time. The goal of this study was to test a variety of electrode configurations for the E2 application to ablate large tissue volumes.

**Materials and methods:**

27 lesions were performed in healthy porcine liver of five female pigs. Four, two and bipolar electrode-arrays were used to deliver various E2 treatment protocols. Liver was harvested approx. 20h after treatment and examined with H&E and Masson’s trichrome staining, and via TUNEL staining for selective specimen.

**Results:**

All animals survived the treatments without complications. With four electrodes, a lesion of up to 35x35x35mm volume can be achieved in less than 30s. The prototype bipolar electrode created lesions of 50x18x18mm volume in less than 10s. Parameters for two-electrode ablations with large exposures encompassing large veins were found to be good in terms of vessel preservation, but not optimal to reliably close the gap between the electrodes.

**Conclusion:**

This study demonstrates the ability to produce large lesions in liver within seconds at lower limits of the E2 parameter space at different electrode configurations. The applicability of E2 for single electrode ablations was demonstrated with bipolar electrodes. Parameters for large 4-electrode ablation volumes were found suitable, while parameters for two electrodes still need optimization. However, since the parameter space of E2 is large, it is possible that for all electrode geometries optimal waveforms and application protocols for specific tissues will emerge with continuing research.

## Introduction

Minimally invasive focal tissue ablation has become an emerging field in modern medicine. The biophysical effects which are employed for tissue ablation by electricity can be divided into thermal and non-thermal techniques. The thermal modalities, which are based on temperature elevation caused by dissipation of electrical energy (Joule heating effect) utilize a variety of electromagnetic frequencies to achieve the effect, including radio frequency [1] and microwave frequency [2]. On the contrary, electrochemical treatment via electrolysis [3] and electroporation-based therapies [4,5,6,7] are based on non-thermal biophysical mechanisms. The effect of electrochemical treatment depends on the generation of electrolysis products via the application of low direct current in the tissue, thus making local changes in pH its main cause of cell death [8]. Because of that and similarly to heat-based therapies, electrochemical treatment does not have preservative properties towards sensitive structures. The advantage of the treatment lies in the application of very low voltages, which reduces instrumentation complexity. However, effective ablation via electrolysis requires relatively high concentrations of electrolysis products. In order to produce sufficient amounts of these products, long treatment times (tens of minutes to hours) are necessary. The biggest drawback is that given the long treatment time, diffusion of the electrolysis products, which can be predicted by considering the electric current applied multiplied by the time of its application, through inhomogeneous tissue and blood transportation lead to an almost unpredictable distribution of electrolytic products. This makes ablation dimensions difficult to plan.

Electroporation is defined as the permeabilization of the cell membrane through the application of pulsed electric fields [9]. The effect on the cell membrane is a function of the electric field strength and pulse properties such as duration, frequency and shape. The decisive advantage of electroporation-based therapies is the fact that they affect only the cells in tissue and spare the extracellular matrix. This can be considered advantageous for many clinical applications, in particular for treatment of tumors near sensitive structures such as blood vessels or nerves [10]. When lower electric fields (below the threshold of approx. 1000-1500V/cm) are employed, reversible electroporation takes place, in which case the cell returns to its original state a few minutes to up to half an hour after the electric field has ceased [11]. This phenomenon can be utilized to transport genetic material [12], drugs [4], dyes [13] and other molecules such as calcium [14] into the cells. Electrochemotherapy (ECT) is the combination of reversible electroporation with chemotherapeutic agents and has been used successfully for tumor ablation in clinical settings [4,6]. The application of higher electric fields results in cell death through a mechanism called Irreversible Electroporation (IRE), where the cells defer to the membrane permeabilization instead of recovering from the treatment without membrane damage [15]. IRE has also gained clinical success [16], especially due to its non-thermal properties and the fact that it does not require the application of drugs. Electroporation-based therapies are much faster than conventional electrochemical treatments. However, a drawback of the IRE procedure is that it requires very high electric field strengths, in the order of 1500 to 3000 V/cm, and the application of hundreds of pulses (e.g. 90 pulses between each electrode for the application in prostate tissue) over up to 30min with strict limitations on distance and parallelism of electrodes. This results in restrictions regarding the resulting lesion size. Additionally, the application requires deep muscle relaxation, as it induces muscle contractions [17]. These muscle contractions may cause significant movement of the electrodes during treatment, resulting in possible complications [18]. This is more likely when the advised number of pulses for clinical applications (usually around 100 per electrode pair) are delivered. The problem of muscle contractions has been partially solved by applying high frequency IRE (HFIRE), however, a small amount of muscle relaxant is often still required [19], and management of high electric field strengths and strict electrode placement rules still apply. Additionally, the high electric fields of above 1500V/cm in IRE treatment almost inevitably spark the gases that get produced during the treatment [20], causing a pressure wave (referred to as discharges, sparks or arcing) with severe acoustic manifestation and mechanical tissue damage. Even when stopped early on, this phenomenon can cause low-impedance situations, resulting in machine failures. In addition to this, the number of pulses typically used in clinical applications substantially lengthen the total procedure time.

Recently it was shown that it is possible to induce ablation by purposefully adding electrolysis to electroporation in a modality that is called electrolytic electroporation (E2) [21]. The E2 ablation technology was developed from basic concept through small animal studies in rats to large animal studies in pigs [21–25]. It has been shown that the combination has advantages over tissue ablation by either electroporation or electrolysis alone [24,25]. One of them is that it requires substantially fewer electric pulses (typically ≤ 5 pulses, depending on ablation size) and at a much lower electric field strength (typically ≤ 1000V/cm) than conventional IRE. The latter is, as explained above, the main reason for most of the challenges of IRE application. At the same time, E2 has similar advantages to IRE, as it is non-thermal and does not require the application of drugs like in ECT, or the application of calcium like in Calcium Electroporation [14]. Potential side effects through these and similar additionally applied substances are therefore not to be accounted for.

**Fig 1:**
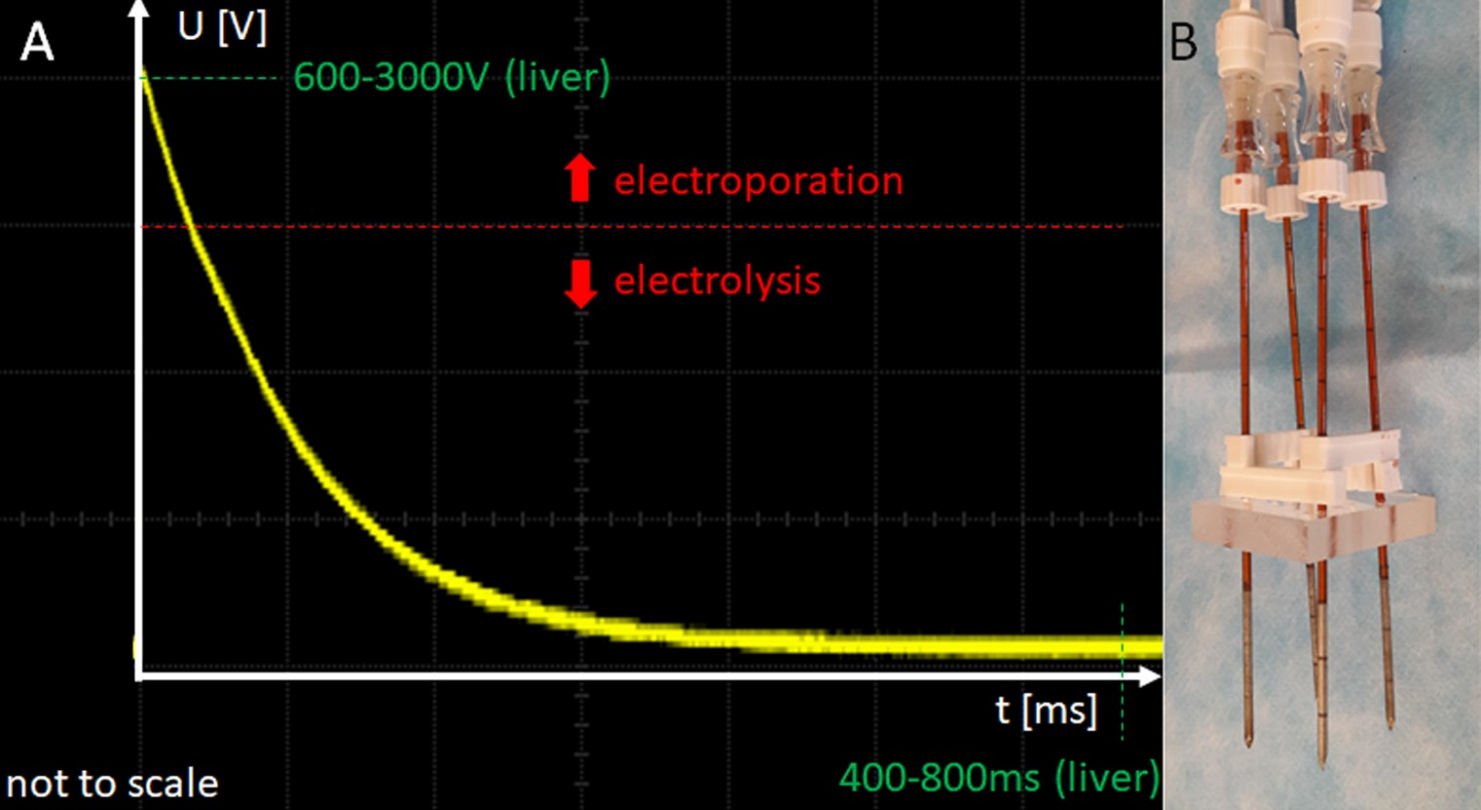
Typical E2 waveform and electrode configuration. A) Outline of a typical E2 waveform in liver tissue. It consists of an initial high-field electroporation phase (600-3000V) and a 400-800ms long, continuous low-voltage tail which transports the produced electrolysis continuously from one electrode to the other (Nernst-Planck type driven diffusion). What parameters are applied depend on the tissue and the electrode geometry. B) A typical 4-electrode configuration, employing 18-gauge electrodes at a distance of 15mm.

The mechanistic explanation of the E2 modality is currently thought to be related to the permeabilization of the cell membrane by electroporation, which makes the cells more sensitive to the products of electrolysis. These can, thereby, enter the cells more easily, causing homeostasis impairment and eventually cell death. Due to the combination with cell permeabilization, the effect can be achieved at a much lower dose than what is required for tissue ablation by electrolysis. Earlier studies on E2 employed waveforms which delivered electrolysis and electroporation separately [22,24,25]. More recently, it was shown that the combination of electroporation and electrolysis can be achieved through the design of an application protocol that delivers electroporation and electrolysis simultaneously [23,26]. The first part of the E2 waveform induces cell membrane permeabilization, while the second part generates the electrolytic products (Fig 1A). This is achieved by discharging several capacitors either sequentially or simultaneously in a judicious way, where key parameters are the initial voltage and the total applied electrical charge. Furthermore, the trailing lower voltage is thought to provide an electrophoretic force to transport the electrolytic products from the electrodes through the treated zone (Nernst-Planck-driven diffusion).

Previous E2 studies have addressed protocols for successful ablation with one electrode pair at relatively short exposure lengths, thus limited ablation volumes. This study was designed to test E2 treatment protocols that can successfully ablate large volumes of tissue employing different electrode geometries. Additionally, large lesions in areas which are usually classified as untreatable with thermal modalities (i.e. near large vessels) were targeted.

## Materials and methods

The experiments were performed in compliance with all ethical and legal rules as stated by the national legislation and the European Union (program number 327). The experimental protocol was reviewed and approved by the Ethics Committee of Fundeni Clinical Institute as well as by the Bucharest Sanitary-Veterinary Authority (no 316). The experimental study was carried out *in vivo* on three 40 kg and two 30 kg breed female pigs, as previously described [27]. In short, after the animals were fasted for 24h, they were medicated with a combination of acepromazine (0.5mg/kg, Vetoquinol S.A., Lure, France) and ketamine (15 mg/kg, Gedeon Richter Plc., Budapest, Hungary), which was injected intramuscularly prior to treatment. Anesthesia was induced intravenously with Propofol at a concentration of 2.5mg/kg and 0.1mg Fentanyl (Chiesi Pharmaceuticals GmbH, Vienna, Austria). After endotracheal intubation was performed, anesthesia was maintained with sevoflurane in 80% O2 (adjusted to 2–2.5% Endtidal sevoflurane, Abbvie, Rome, Italy). In case of postoperative pain, the animals were treated with morphine (Zentiva S.A., Bucharest, Romania) at a concentration of 0.1 mg/kg, applied intramuscularly, and ketoprofen 1 mg/kg (S.C. Terapia S.A., Cluj-Napoca, Romania). Cefazolin 25 mg/kg (Biochemie GmbH, Kundl, Austria) was applied intravenously in 2h intervals. The pigs were placed in a ventral side-up position, and the liver was exposed with an upper midline incision continued with a right transverse incision. The treatment was delivered using 3 types of electrodes: 1) 18-gauge or 16-gauge stainless steel needle-type electrode with a variable exposed length of 1-4cm exposed treatment length (Inter Science GmbH, Lucerne, Switzerland); 2) A 13-gauge stainless steel needle-type electrode (IGEA, Carpi, Italy); 3) A simple, custom-made bipolar electrode, employing 2 shifted 18-gauge electrodes with an isolated gap between. All treatments with their treatment parameters are listed in Table 1. All sets of parameters were tested a minimum of 2 times in healthy pig liver. Total number of lesions was 27.

**Table 1:** Relevant parameters for the experiments which were conducted in this study.

# of electrodes	Distance /mm	Exposure /mm	Capacitance /uF	Electrode diameter /G	Voltage /V	Waveform protocol	Lesion size and uniformity	Figure
4	15	30	293	18	1400, 1000	6+4*	39x37x35mm, UA	2
4	15	20	193	18	1200	6	Not bridged	3
4	15	30	293	16	1400, 1000	6+4*	26x26x35mm, UA	S1 Fig
2	20	30	293	18	1500	2	30x30mm, UA	4
2	20	30	293	13	1600, 1500	2	Not bridged	5
2	15	30	193	13	1500	2	35x21mm, UA	S2 Fig
2	angular	30	293	13	1000, 1200		15x20mm, UA	S3 Fig
Bipolar	15	10	293	18	1200, 1100, 1000	3	50x13mm, UA	6
Bipolar	15	15	193	18	1200	3	Not bridged	S4 Fig

* in these cases, a total of 10 waveforms were applied, where the first 6 were between all electrode pairs at 1400V, and the last 4 were between the peripheral electrode pairs at 1000V.

UA = uniformly ablated treatment area.

In all cases the electrodes were placed in the liver under ultrasound guidance (Hi Vision Preirus Ultrasound device, Hitachi Medical Systems, Wiesbaden, Germany). In all experiments except one, custom made grids and spacer were used to stabilize the electrode array and ensure distance and parallelism between electrodes (Fig 1B). In the one exception, explicitly non-parallel behavior was tested. The oscilloscope (Owon SmartDS Oscilloscope, Fujian Lilliput Optoelectronics Technology Co., Ltd, Industrial Zhangzhou, China) trace in Fig 1A illustrates a typical E2 waveform, which is made by the first part of the E2 wave that is primarily designed to induce electroporation, followed by the part to induce electrolysis. the E2 waveforms were the product of free discharge of a capacitor (of specific capacitance and charged to a specific voltage) across a pair of electrodes in tissue.

After the procedure, the pigs were kept alive for 18–23 hours, after which the liver was harvested and the animal was sacrificed using KCl 7.45% 1 ml/kg (B. Braun, Melsungen, Germany). The liver harvesting procedure was carried out under general anesthesia and is described in detail in [27]. After liver harvest, it was immediately perfused with saline solution and a 10% formalin fixative was perfused in the same way for approx. 10min. All lesions were cut out with normal surrounding parenchyma and stored in formalin solution. The tissue was then bread loafed and put in cassettes, which were routinely handled with 10% phosphate buffered formalin for 8–10 hours, photographed and embedded in paraffin blocks. For microscopic evaluation, 3μm sections were cut from each paraffin block and stained either with hematoxylin & eosin or Masson’s trichrome staining. TUNEL staining was only performed in selected slides. The staining and scanning was performed by a pathology service provider (HistoWiz Inc, NY 11226 US), as per their tissue preparation protocol for sectioning and staining. Whole slide scanning (up to 40x magnification) was performed completed with an Aperio AT2 (Leica Biosystems). Microscopical analysis was performed separately by two scientists trained in the field of liver tissue ablation. At least three sites per slide in the area of the electrode, in the middle between the electrodes, and the border between treated and untreated areas (if applicable) were reviewed and analyzed. Additionally, at least two vessels enclosed in the treatment area were evaluated per slide.

## Results and discussion

### E2 ablation with a four-electrode array

**Fig 2:**
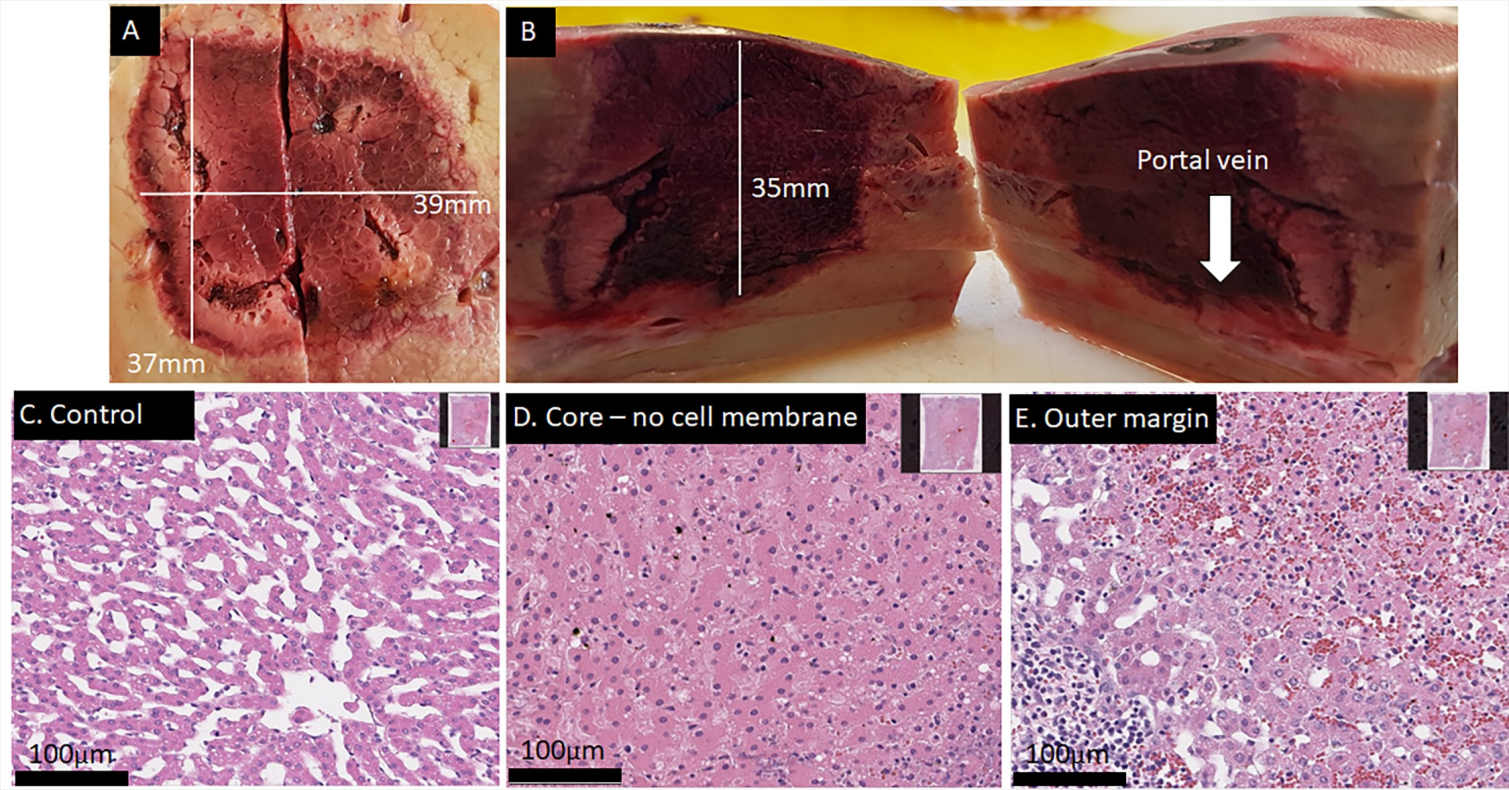
Result of a successful 4-electrode array ablation using a total of 10 waveforms in less than 30s total delivery and recharge time. Liver harvest was 18h post treatment. The lesions size is approx. 39x37x35mm. (A+B) Gross pathology and lesion dimensions. (C) H&E staining of an untreated area. (D) H&E staining of an area in the middle of the lesion (core region). Sample shows cell death throughout, with a mixture of severe apoptosis and complete hepatocellular necrosis. (E) H&E staining of the outer margin with a sharp transition of completely dead tissue (upper right corner) to apparently unaffected (or already regenerated) liver tissue (lower left corner).

Fig 2 shows macroscopic and microscopic results obtained with four electrodes. The electrodes were in a parallel square alignment 15mm apart, held by a spacer with 30mm stainless steel exposure length. The electrodes had a diameter of 18G. Pulse protocol included that each pair had a potential difference of 1400V and 293μF. To add more electrolysis, another pulse round (without the diagonals) was delivered with the same capacitance but a decreased applied voltage of 1000 V. Fig 2 in conjunction with the histopathology shows that this protocol produced a continuous lesion of approximately 35x35x35mm. The total deliver/recharge time was less than 30 seconds, with a 30 second delay between the two rounds due to manual export of oscilloscope data. Similarly, the lesion shown in S1 Fig followed the identical protocol with different electrodes (16-gauge) and displays similar convincing results with slightly smaller outer dimensions, most likely because appx. 20% less total charge was delivered into the same volume because other electrodes were used (compare Fig 2 and S1 Fig). For reference, an IRE application with the commercially available generator (NanoKnife, Angiodynamics, NY, USA) and the recommended settings would have required more than 1300s of treatment time and a manual push-forward of all electrodes to achieve the same result. From comparison it is obvious that the outcome of the treatment is repeatable and a large complete ablation can be produced with a four-electrode array.

**Fig 3:**
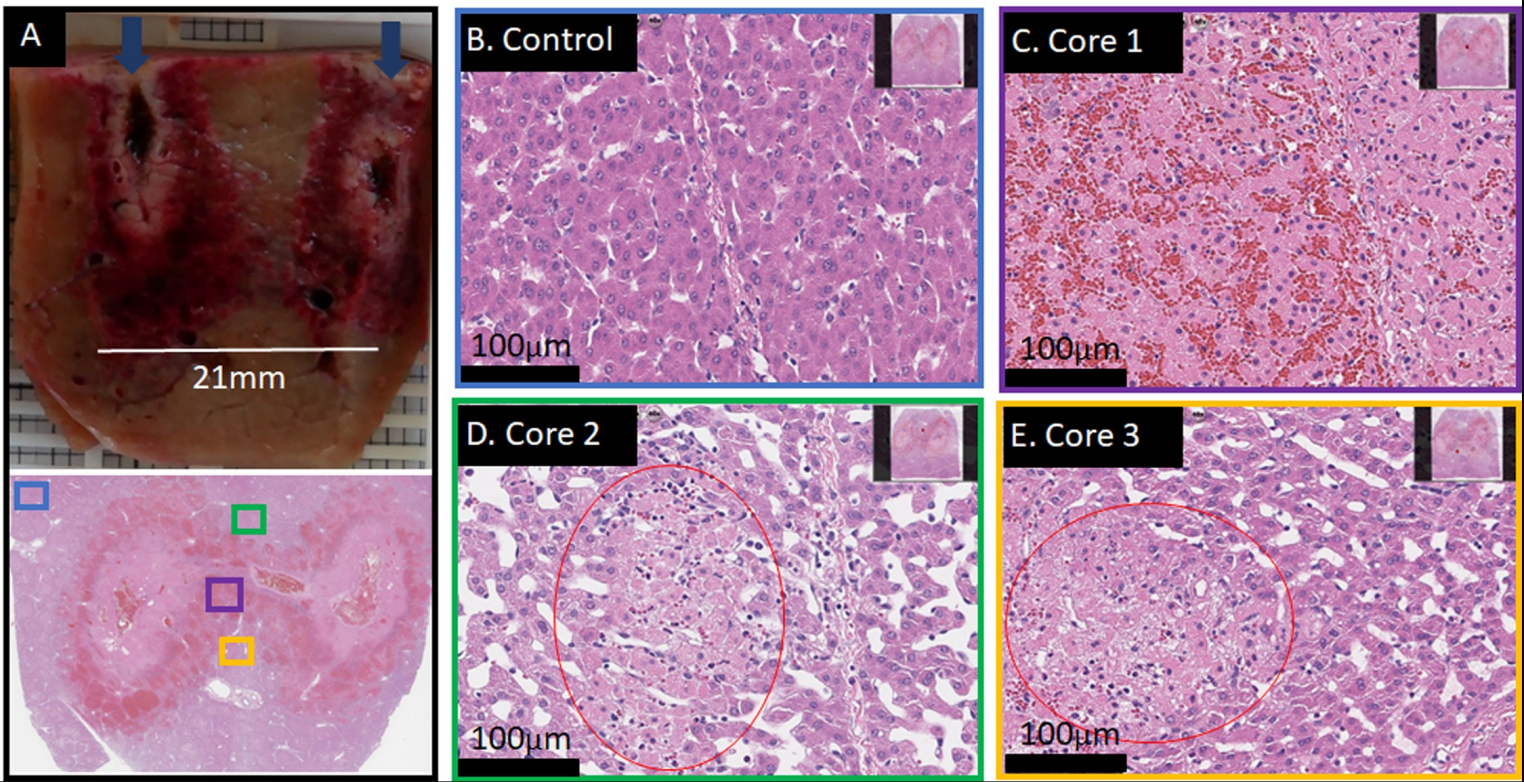
A 4-electrode array ablation with insufficient treatment outcome. Treatment parameters the same as shown in Fig 2. Liver harvest was 22h post treatment. Six waveforms at 1200V (every pair once) were delivered in total. (A) Gross pathological cut between two electrodes of a 4-electrode array ablation. Only within a thin path which connects the two electrodes, complete cell death is found. Dark blue arrows indicate direction of electrode track. (B) Control, H&E stained at 20x magnification. (C) H&E staining of the sample at 20x magnification, illustrating the purple area shown in A. Within the thin path, cells are completely dead. (D and E) H&E stained samples at 20x magnification of the outer areas (green and yellow boxes in A, respectively) at the core of the lesion, which are effected and show “patches” of dead cells (red circles) throughout.

Fig 3 shows only partial ablation between the electrodes. 1200V were employed for the same geometry as described in Fig 2, but instead of 6+4 pulses, only 6 pulses (all electrode pair permutations) were applied. Both the maximal field as well as the total charge (per volume tissue) are lower than in the cases presented in Fig 2 and S1 Fig. This resulted in too little electrolytic species per volume tissue and a field that was too low for reliable reversible electroporation throughout the treatment field. The “hourglass” shape is typical for electrical field-based procedures with insufficiently high parameters. While some areas appear completely ablated (Fig 3C), there are areas in the middle of the ablation zone which were only partially ablated (Fig 3D and 3E). The observation of this effect might be a function of time, as regeneration processes in liver start quickly. Nevertheless, complete cell-death, as desirable for most non-drug assisted cancer treatments, appears not to be guaranteed with this treatment protocol. This demonstrates that the parameters used here are slightly below the lower threshold of complete ablation.

**Fig 4:**
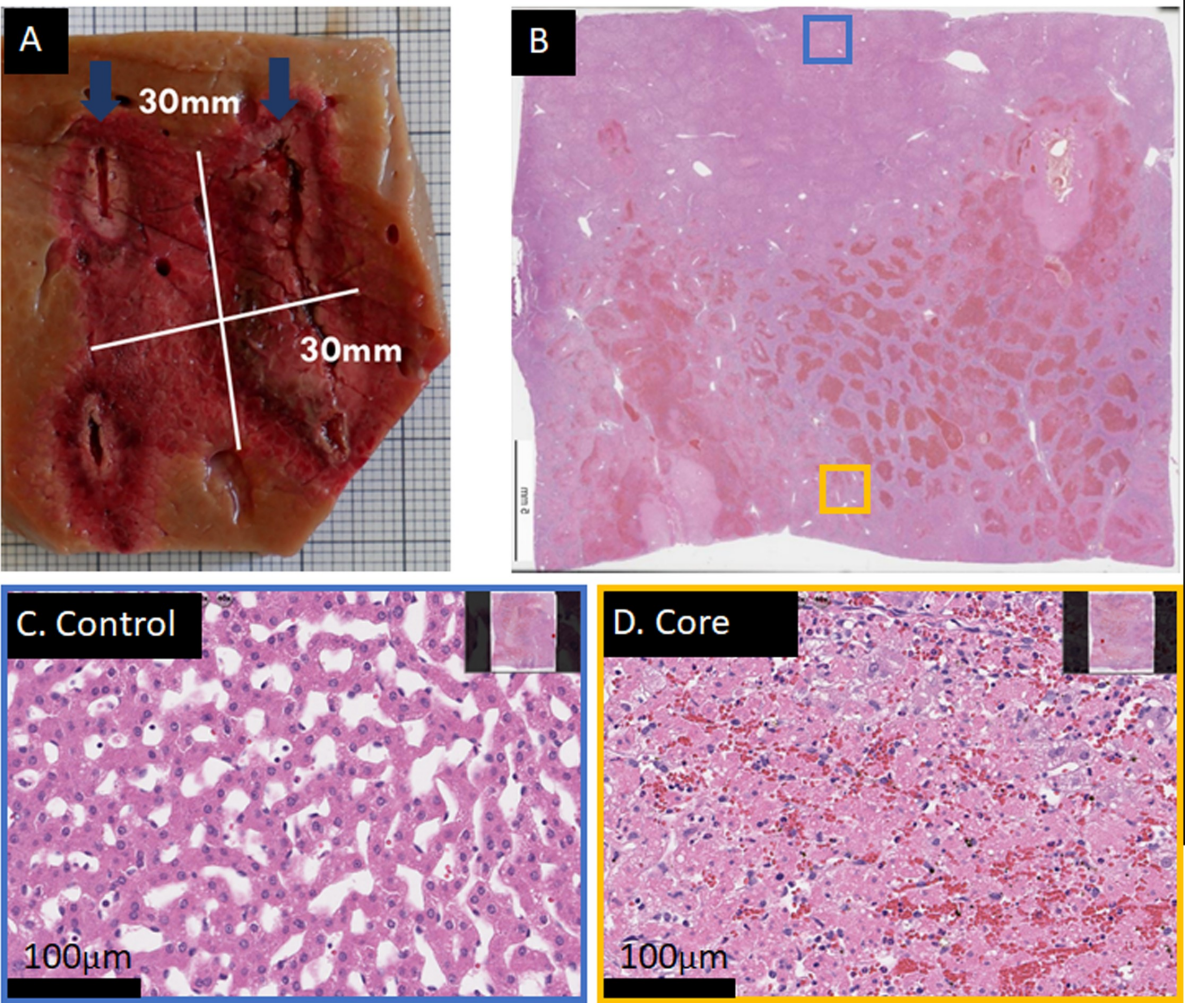
Successful 2-electrode ablation. There was a distance of 20mm between the electrodes, with an exposed length of 30mm and custom-made electrodes of 18G diameter, using 2 waveforms of 1500V peak voltage. Total time required was approx. 5s. Liver was harvested 23h post treatment. (A) The outline of the macroscopic coronal cut, showing the area between the electrodes. Dark blue arrows indicate direction of electrode track. (B) H&E staining of the upper part of the lesion. (C) H&E stained control area at 20x magnification, blue insert in B. (C) H&E stained area at 20x magnification from the core region (yellow insert in B). Severe cell death is found throughout the lesion. Note that the sample was cut out of plane, hence there is no perfect match between macroscopic and microscopic images.

Comparable IRE treatments of pig liver were performed and are described in [15]. In their experimental setting, four electrodes were arranged in a 1.5 cm square, with an exposure of 1 cm, in which a total of 8 pulses, 100μs long and a voltage of 2500V were delivered to successfully ablate tissue between all electrodes. In comparison, the results in Figs 3 and 4 were obtained with 1400V and 10 waveforms. As the applied current in E2 is much lower than in IRE, tissue with 3cm exposure length or longer can be achieved. With today’s recommendations for clinical IRE, an IRE treatment that achieves the same result would need an application of 540 pulses at 2250V.

### E2 ablation with a two electrode array

An example of a complete ablation between two electrodes with E2 is shown in Fig 4 and S2 Fig. The lesion in Fig 4 was obtained with two waveforms at 1500V and 293μF, at a distance of 2cm and exposure length of 3cm. For the lesion in S2 Fig, the same parameters were applied, however at a distance of 2cm. Here, the damage appears to show less signs of necrosis and structural loss compared to S2 Fig, possibly due to higher distance at same voltage and charge. The results in Fig 4 and S2 Fig show that it is possible to obtain complete ablation with two electrode needles with only the delivery of two waveforms at field strengths equal to what is used in ECT. In contrast, a similar procedure with IRE would require 2500V and 180 pulses with a delivery time of appx. 220 seconds, not including the time for manual push-forward procedure due to the limited maximum exposure length in IRE application.

**Fig 5:**
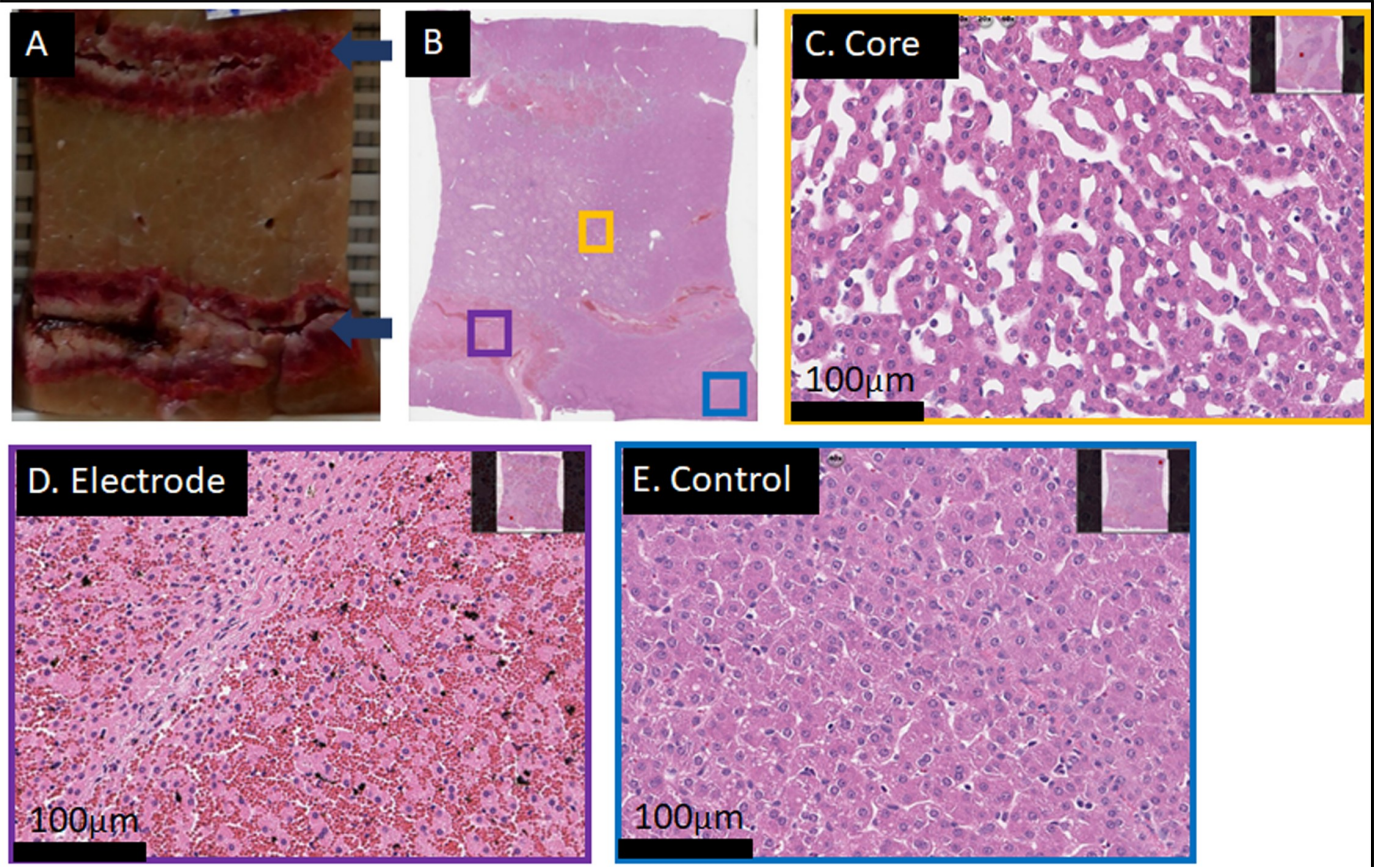
Example of a non-bridged 2-electrode ablation. Two waveforms at 1600V and 1500V peak voltage were applied, delivered with 13G diameter electrodes at 20mm distance and 30mm exposure length. Total time required was approximately 5s, however the waveforms were cut off early by the software, leading to incomplete discharge and hence incomplete delivery and distribution of electrolysis. Liver harvest was 22h post treatment. (A) Macroscopic image of the lesion, which shows healthy looking tissue between the electrodes (electrode track is visible as deeply darkened tissue, direction is indicated in dark blue arrows). (B) H&E staining of the entire lesion. (C) H&E stained tissue sample at 20x magnification from the core region (yellow insert in B). Sinusoids and cells appear affected, but not dead. (D) H&E stained tissue shows complete cell death and disrupted cellular infrastructure at the electrodes at 20x magnification (purple insert in B). (E) H&E stained control area at 20x magnification, blue insert in B.

An example of improper E2 parameters which will result in a non-bridged lesion is shown in Fig 5. A possible explanation in this case is that the applied waveforms were cut-off too early by the software and not completely delivered, hence about 30% less charge per volume than in the lesion shown in Fig 4 was applied. This may identify as a lower E2 margin for two-electrode arrays.

### E2 ablation with non-parallel electrodes

The application of two electrodes, in which the electrodes are not parallel and at one point come very close to each other, was tested in another experiment. We included this experiment to see if E2 application has an advantage over IRE in cases where electrodes are not strictly parallel. Typically, in IRE procedures such an application will cause rapid discharge and resulting shut-down of the device. The results of the experiment are shown in S3 Fig. A complete ablation of the area between the active parts of the electrodes and beyond was feasible. Microscopical results at different relevant sites (S3C–S3E Fig) compared to control (S3B Fig) show that the anticipated lesion was fully ablated throughout. A gradient of cell death can be seen, starting from the convergence (left in S3A Fig) of severe thermal necrosis to complete hepatocellular necrosis to severe apoptosis at the most diverged parts (S3C Fig). In the areas of thermal necrosis, the vessels were damaged, as to be expected (S3D and S3E Fig). No discharge (ignited plasma formation) was noted. A geometry like this is not possible with IRE pulses because the required high field allows little wiggle-room, resulting in gaping plasma formations between converging electrodes [20]. An ignited plasma spreads due to its very low resistance. If not shut down immediately, immense energies can be pumped into the tissue in microseconds. As E2 is further below this plasma ignition threshold, convergence of electrodes is less of a problem. However, as to be expected, joule heating remains more prevalent in these high-field areas. Hence the mode of death in this area shifted more towards thermal induced cell death.

### E2 ablation with a bipolar electrode

**Fig 6:**
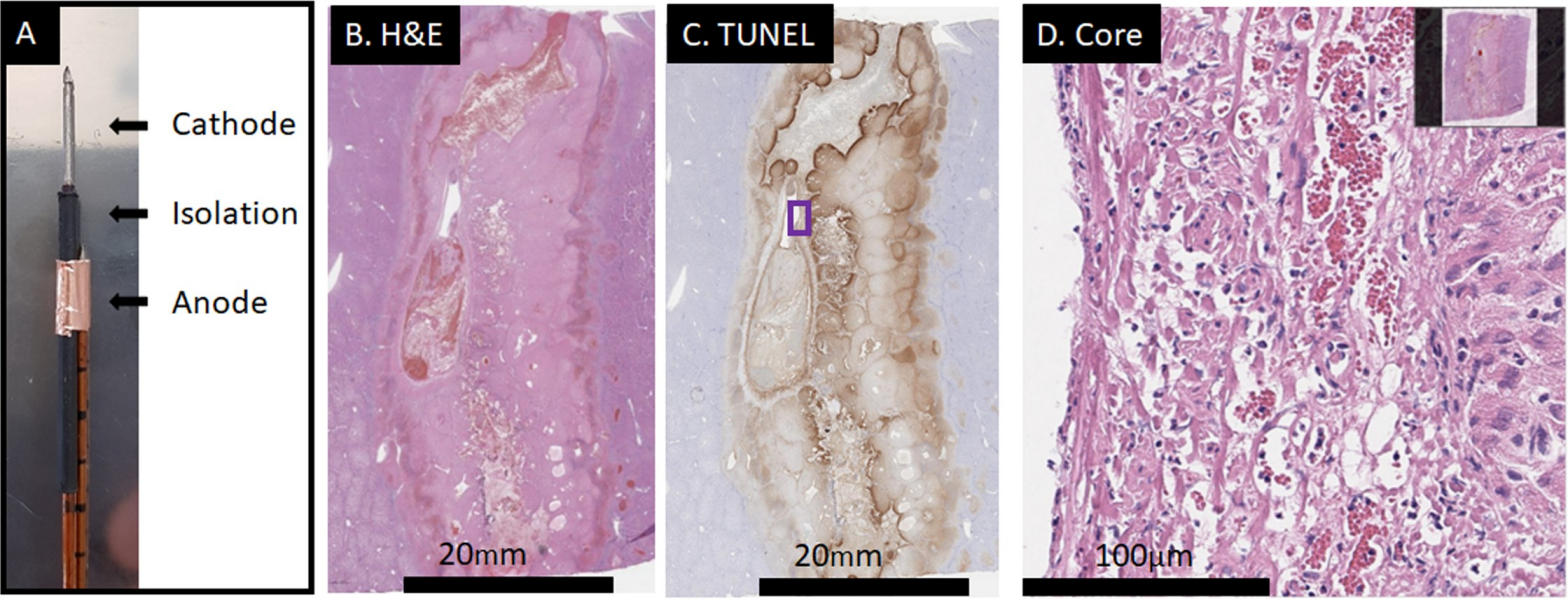
A successful ablation with a bipolar electrode. Applied were three E2 waveforms at 1200, 1100 and 1000V peak voltage and 293μF capacitance. Liver harvest was 18h post treatment. (A) The electrode prototype had lengths of 10-10-15mm for anode-isolation-cathode, respectively. (B) H&E staining of the entire lesion. (C) TUNEL-staining of the entire lesion. Both stains show a clean, closed lesion with a mixture of necrosis and significant apoptosis towards the margins.

Fig 6 and S4 Fig show results for the application of the prototype bipolar electrode. Fig 6 shows the application in which the lesion closed between the two electrodes. In this experiment, three waveforms at voltages of 1200, 1100 and 1000V, respectively, and at 293μF were delivered in a total of 9 seconds. In the experiment shown in S4 Fig, the same pulse protocol was applied, but only 193μF was utilized. In addition, the isolated distance of anode and cathode was higher with 1.5cm instead of 1cm (Table 1). It is evident that much less electrolysis was delivered in the incomplete ablation.

## Conclusion

A study was performed on the liver of five pigs to evaluate E2 applications for large lesions. The goal of the study was to develop a better understanding of the effects of E2 parameters on tissue ablation with the boundary conditions of fast, large and vessel-preserving ablations, employing different electrode arrays that are often used in clinical settings. Our results demonstrate the ability to produce large continuous lesions with a 4-electrode array, a 2-electrode array and a bipolar electrode encompassing large vessels in a majority of the lesions. Lesions as large as 35x35x35mm were achieved without requiring replacement of the electrodes. No major bleeding occurred, no clinically significant adverse events occurred and no subject died during treatment or observation time. Similar to other electroporation-based treatments, parameters that are below the effective threshold will lead to lesions that do not close. This was especially evident with the parameters chosen for the two-electrode experiments. These were all at the lower thresholds, mainly due to the desire to investigate E2 application without deep muscle relaxant. The results obtained with the two-electrode array demonstrate the need to provide optimal treatment parameters for E2 in clinical settings. However, since the domain of E2 parameters is large, it is possible that different optimal waveforms and protocols for specific tissues and applications will emerge with continuing research.

## Supporting information

S1 Fig Result of a successful 4-electrode array ablation using a total of 10 waveforms. Analog ablation to the one shown in Fig 2. While the electrical and geometrical parameters were identical, E2 electrodes of 16G diameter were used, which allowed for approx. 20% less charge to be delivered in the same amount of time. Liver harvest was 22h post treatment. (A) Gross pathology of the lesion, showing approximate lesion dimensions of 26x26x35mm. (B) H&E stained control area at 20x magnification. (C) H&E stained tissue sample at 20x magnification from the core region, showing complete ablation, congruent with macroscopic impression. (D) H&E stained tissue sample at 10x magnification from the core region, showing a large vessel. Cellular elements of vessels appear ablated. However, the fibers support the tissue infrastructure sufficiently to maintain its function.
(TIF)

S2 Fig A successful 2-electrode lesion performed at 1500V in less than 5 seconds. The parameters were 2 waveforms at 1500V, 15mm distance, 30mm exposure length with 13G diameter electrodes. Liver harvest was 22h post treatment. (A) Macroscopic lesion (top), and Masson’s Trichrome staining of the entire lesion (bottom). Dark blue arrows indicate direction of electrode track. (B) H&E stained control area at 4x magnification. (C) H&E stained tissue sample at 4x magnification from the core region, showing complete cell death throughout the area. (D) Masson’s trichrome staining at 4x magnification of an untreated area. (E) Masson’s trichrome staining at 4x magnification of an area in the middle of the treatment field (yellow insert in A) were several vessels were present. All vessels showed an intact vessel structure, while all surrounding cells were ablated.
(TIF)

S3 Fig A 2-electrode lesion with non-parallel free-hand insertion. The parameters were 1000V and 1200V peak waveform voltage respectively, using 13G diameter electrodes with 30mm exposed length at 293μF capacitance. The distance between the electrodes was estimated using ultrasound measurement. Liver harvest was 22h post treatment. (A) Gross macroscopic image of the lesion (top) and H&E stained slide of the whole lesion (bottom). Dark blue arrows indicate direction of electrode track. (B) H&E stained control area at 20x magnification (blue insert in A). (C) H&E stained tissue sample at 20x magnification from the core region (green insert in A), showing complete cell death throughout the area. (D) Masson’s trichrome staining of a vessel which was enclosed in the treatment area (yellow insert in A). Thermal necrosis is apparent, which the vessel was severely affected by. (E) Masson’s trichrome staining of another vessel which was close to where the electrode tips almost touched (purple insert in A). Thermal necrosis has affected the vessel infrastructure.
(TIF)

S4 Fig Non-continuous lesion of a bipolar electrode. 15-15-15mm were used as anode-isolation-cathode lengths, respectively, employing 3x1200V peak voltage for the waveforms and 193μF charge. Image shows the measurements of the lesion.
(TIF)
