# Supplementary figures and images for: The combination of electroporation and electrolysis (E2) employing different electrode arrays for ablation of large tissue volumes
Source: PLoS One. 2019 Aug 22;14(8):e0221393. doi: 10.1371/journal.pone.0221393 (PMC6705851; doi:10.1371/journal.pone.0221393)

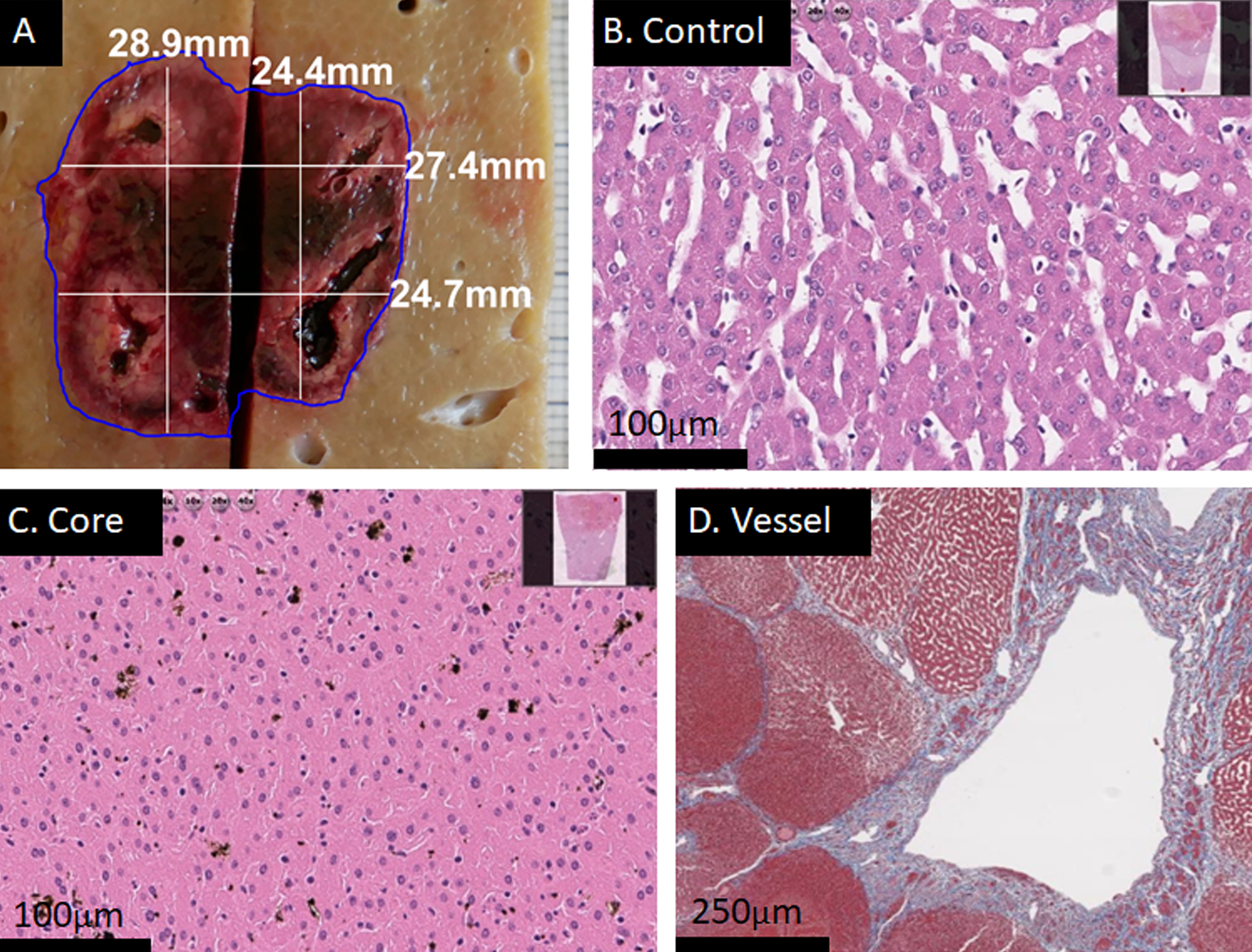

Supplement: S1 Fig — Analog ablation to the one shown in Fig 2. While the electrical and geometrical parameters were identical, E2 electrodes of 16G diameter were used, which allowed for approx. 20% less charge to be delivered in the same amount of time. Liver harvest was 22h post treatment. (A) Gross pathology of the lesion, showing approximate lesion dimensions of 26x26x35mm. (B) H&E stained control area at 20x magnification. (C) H&E stained tissue sample at 20x magnification from the core region, showing complete ablation, congruent with macroscopic impression. (D) H&E stained tissue sample at 10x magnification from the core region, showing a large vessel. Cellular elements of vessels appear ablated. However, the fibers support the tissue infrastructure sufficiently to maintain its function. (TIF) [file pone.0221393.s001.tif]

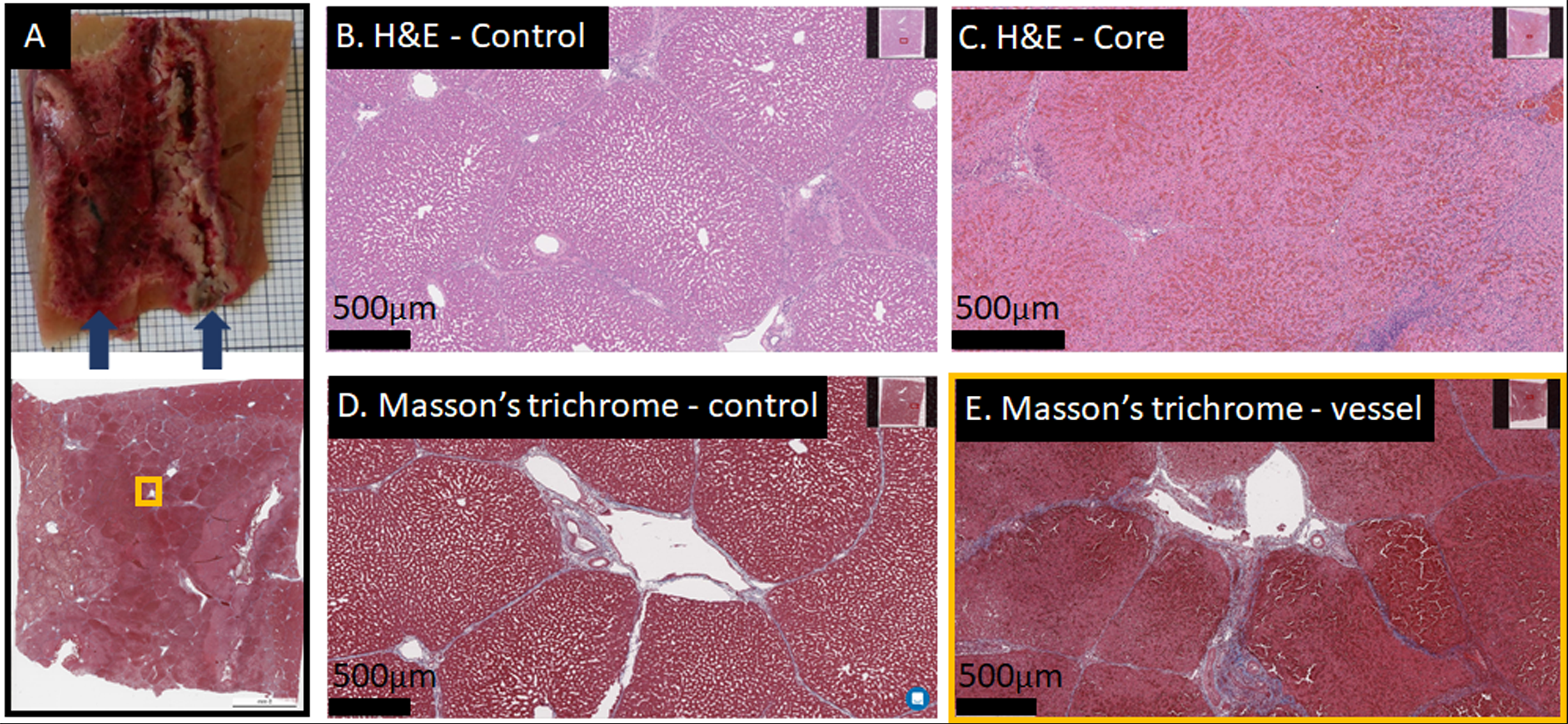

Supplement: S2 Fig — The parameters were 2 waveforms at 1500V, 15mm distance, 30mm exposure length with 13G diameter electrodes. Liver harvest was 22h post treatment. (A) Macroscopic lesion (top), and Masson’s Trichrome staining of the entire lesion (bottom). Dark blue arrows indicate direction of electrode track. (B) H&E stained control area at 4x magnification. (C) H&E stained tissue sample at 4x magnification from the core region, showing complete cell death throughout the area. (D) Masson’s trichrome staining at 4x magnification of an untreated area. (E) Masson’s trichrome staining at 4x magnification of an area in the middle of the treatment field (yellow insert in A) were several vessels were present. All vessels showed an intact vessel structure, while all surrounding cells were ablated. (TIF) [file pone.0221393.s002.tif]

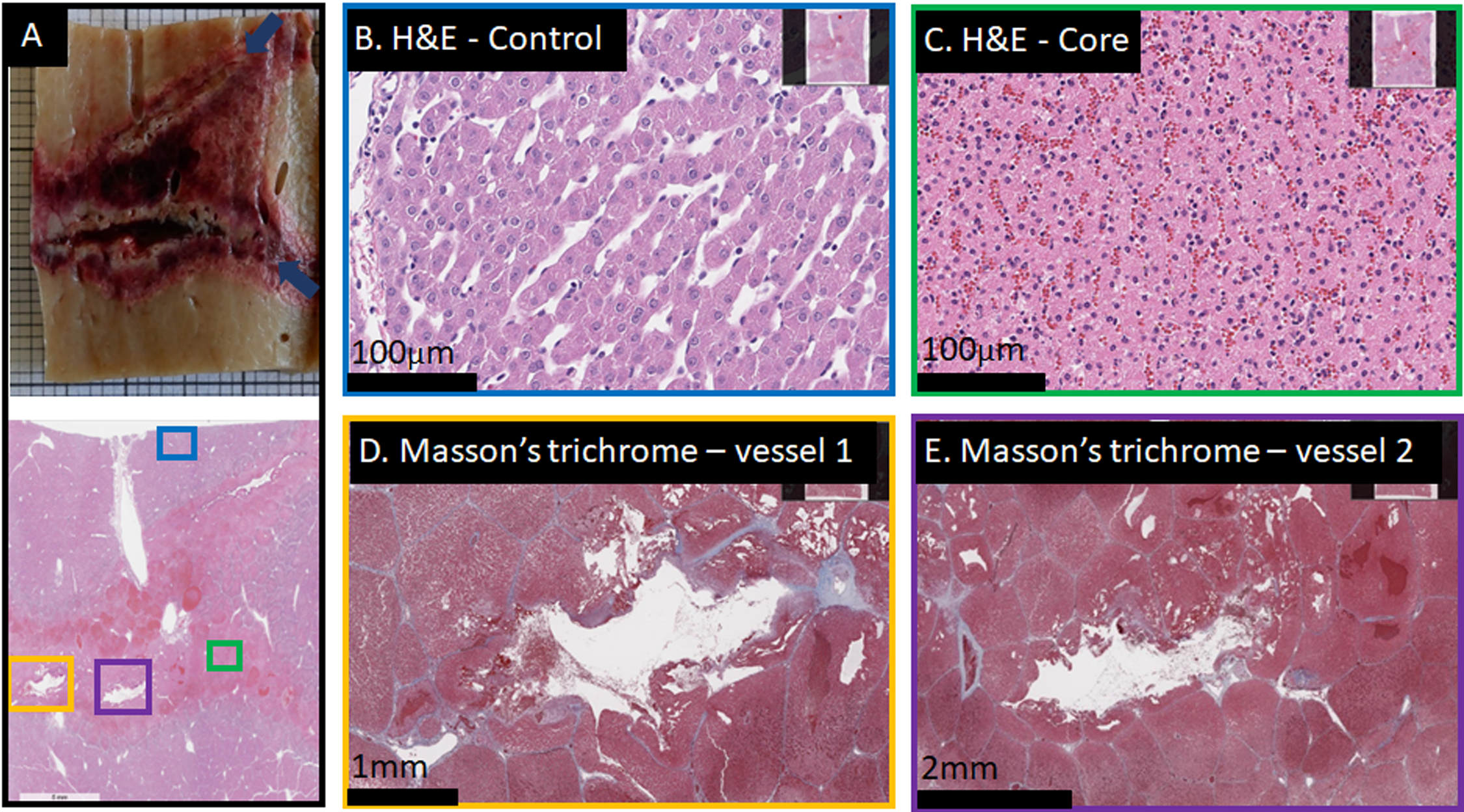

Supplement: S3 Fig — The parameters were 1000V and 1200V peak waveform voltage respectively, using 13G diameter electrodes with 30mm exposed length at 293μF capacitance. The distance between the electrodes was estimated using ultrasound measurement. Liver harvest was 22h post treatment. (A) Gross macroscopic image of the lesion (top) and H&E stained slide of the whole lesion (bottom). Dark blue arrows indicate direction of electrode track. (B) H&E stained control area at 20x magnification (blue insert in A). (C) H&E stained tissue sample at 20x magnification from the core region (green insert in A), showing complete cell death throughout the area. (D) Masson’s trichrome staining of a vessel which was enclosed in the treatment area (yellow insert in A). Thermal necrosis is apparent, which the vessel was severely affected by. (E) Masson’s trichrome staining of another vessel which was close to where the electrode tips almost touched (purple insert in A). Thermal necrosis has affected the vessel infrastructure. (TIF) [file pone.0221393.s003.tif]

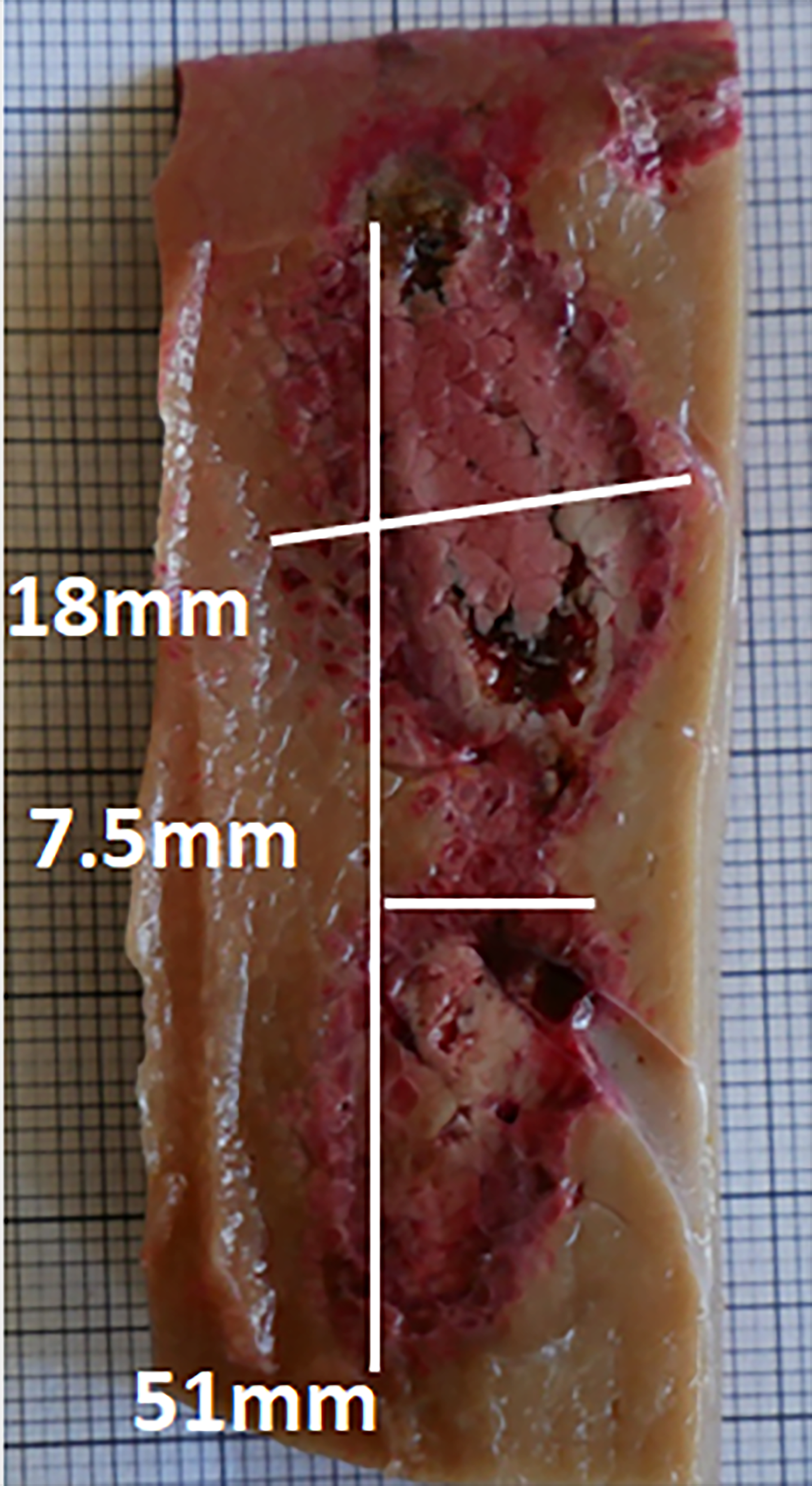

Supplement: S4 Fig — 15-15-15mm were used as anode-isolation-cathode lengths, respectively, employing 3x1200V peak voltage for the waveforms and 193μF charge. Image shows the measurements of the lesion. (TIF) [file pone.0221393.s004.tif]
